# The Royal Naval Medical Service

**Published:** 1920-09-04

**Authors:** 


### THE ROYAL NAVAL MEDICAL SERVICE.

Within the last few years, apart from the stimulus of the war period, a remarkable campaign of reconstruction and improved equipment has been carried out in the Royal Naval Medical Service, and those entering it now at the increased rates of pay and in the face of decidedly enlarged opportunities of individual professional achievement will reap the full benefit of these changes. On another page we give full details of the conditions of service, pensions and general duties, but in this short note we wish simply to testify from personal experience to the excellence which characterised the war work of the R.N.M.S. It is true that at no time did the service encounter such severe pressure as befell their colleagues in the army, but there was plenty of work to be done, both medical and administrative, both ashore and afloat, and that, more often than not, at the least expected moments. Excellence of equipment and an inflexible routine (most irksome, indeed, until experience taught us its remarkable value) proved equal not only to the demands of emergency but allowed of much invaluable observational work on the peculiarities of naval medicine; and of this the pages of the R.N. Medical Journal, during and after the war, provide many examples.

The customary life as a surgeon-lieutenant R.N., consists of rotational spells of some months' duty (*a*) in the big naval hospitals, establishments comparing more than favourably with any institution in the world, (*b*) as medical officer to sea-going ships of the R.N., and (*c*) shore duties as M.O. in a variety of naval establishments either at home or abroad. As far as possible, the times and localities of these appointments are distributed so that each officer has in the long run his fair share of desirable (and undesirable) posts, and few indeed have any real cause for complaint. For a young man who would see the world, learn the true meaning of the word "efficiency," and spend some years in company with one of the finest sets of men he could ever wish to meet, we confidently recommend service with His Majesty's Navy.

Successful candidates are first appointed acting surgeon-lieutenants, and then pass on to a course of training, the first two months of which are passed at the Naval Medical School at Greenwich, and the remainder at Haslar, where special attention is given to the naval aspects of hygiene, recruiting, physical training, diving and submarine work, radiography, anæsthetics, dentistry, etc. A recent development arises with the permanent recognition of forty-six officers out of the total *personnel* who will be allowed to carry on duty as medical and surgical specialists in various necessary branches and according to their qualifications and professional attainments.

